# Indiscriminate antibiotic prescribing for nonspecific symptoms perpetuates gender-based healthcare inequities

**DOI:** 10.1016/j.xagr.2025.100524

**Published:** 2025-06-02

**Authors:** Alec Szlachta-McGinn, Lynn Stothers, A. Lenore Ackerman

**Affiliations:** 1 Division of Urogynecology and Reconstructive Pelvic Surgery, Department of Obstetrics and Gynecology, University of Illinois Chicago, Chicago, IL (Szlachta-McGinn); 2 Division of Urogynecology and Reconstructive Pelvic Surgery, Department of Urology, University of California, Los Angeles, Los Angeles, CA (Stothers and Ackerman)

**Keywords:** antibiotic stewardship, healthcare inequities, urinary tract infection

## Abstract

Urinary tract infection is among the most common bacterial infection among adults, and women are significantly more likely to experience urinary tract infection than men. The prevalence of urinary tract infection in women increases with age, as does the prevalence of noninfectious lower urinary tract symptoms and asymptomatic bacterial colonization of the urinary tract, known as asymptomatic bacteriuria. Distinguishing among these 3 entities is challenging without a complete clinical evaluation, including history, physical examination, and urine culture data. Existing literature demonstrates high misclassification of nonspecific symptoms, such as urinary tract infection, among women. In addition, less than one-fifth of diagnoses meet evidence-based criteria for urinary tract infection. Therefore, women are burdened by several healthcare inequities, including delays in care for potentially life-threatening conditions, antibiotic resistance and antibiotic-associated adverse events because of overreliance on antibiotics for noninfectious symptoms, and distrust of medical care. Profit-driven pressures imposed by our healthcare system, which reward providers for increasing clinical volume at the expense of quality patient-provider interactions, are a major culprit driving these inequities. In addition, lack of provider knowledge regarding urinary tract infection–confusable diagnoses, discomfort with pelvic examinations, and inappropriate use of automated question-based algorithms to diagnose and treat urinary tract infection are to blame. An evidence-based approach incorporating a focused history and physical examination that is concordant with the patient’s chief complaint in addition to urine culture data only in cases of suspected urinary tract infection is the only way to reduce urinary tract infection–related healthcare inequities unfairly confronting women.

## Inequities in women’s health endure

A 63-year-old patient with uncomplicated overactive bladder controlled on medications stated at the start of her televisit, “I went into the ER because I found some blood in my stools and I’m so glad I did, cause they told me I really just had a UTI!” Such stories are commonplace in our clinic. Women with “urinary tract infections [UTIs]” provide similar commentary—their care focuses on treating any abnormal testing instead of addressing their presenting complaints, they are not examined, and their symptoms can frequently be written off as UTI, particularly if they have pain or discomfort anywhere below the waist.

A 70-year-old patient with multiple sclerosis presented to our clinic for urgent follow-up after being discharged from a local emergency department (ED) near her home. The patient had recently called 9-1-1 with heart palpitations and associated lightheadedness, leading to a near-syncopal episode. In the ED, the patient was tachycardic and hypotensive. The patient said, “They told me I had a positive urinalysis and bacteria in my urine, so they gave me antibiotics for a urine infection and sent me home.” The patient did not have dysuria, bladder pain, or other signs of infection. We performed an electrocardiogram, which revealed a new cardiac arrhythmia as the likely cause of the new symptoms, prompting urgent cardiology evaluation.

A 74-year-old woman presented for evaluation of recurrent UTIs. In the past 3 months, the patient has been to the ED 4 times, each time for a fall from a standing position. No urine cultures were performed. Urinalyses exhibited only trace leukocyte esterase without nitrites. The patient had described new urinary incontinence but no dysuria or bladder pain. However, the patient’s symptoms included progressive balance and gait issues, memory problems, mental confusion, headaches, and vision changes. With each subsequent visit, stronger and longer-duration antibiotics were prescribed without any change in the patient’s condition. After our consultation, we obtained a head computed tomography scan that revealed hydrocephalus. Shortly thereafter, the patient underwent shunt placement with significant improvement in these symptoms.

In addition, premenopausal women are not immune to such inequities in care. A 44-year-old woman with regular menses presented for evaluation of recurrent UTI. The patient reported that she had received treatment for at least 6 infections in the past year. The patient’s only symptom was fluctuating chronic dysuria. The patient did not report urinary urgency, frequency, suprapubic pain, or any other symptoms of UTI. The patient’s urine cultures were repeatedly negative. However, the patient received several courses of antibiotics with minimal or no resolution of her symptoms. On a more thorough evaluation of the patient’s symptoms, the patient reported that the burning sensation with urination was located on her vulvar skin. Pelvic examination revealed skin changes in the labia minora consistent with lichen sclerosus, which biopsy confirmed. The patient responded well to a topical steroid ointment, and her symptoms resolved.

## Women’s symptoms are frequently dismissed or misdiagnosed

In the United States and elsewhere, women’s health is often marginalized. In clinical medicine, women are more likely to die after myocardial infarction than men, suffer from untreated chronic illness, and experience delays in care even after diagnosis. In clinical research, women have been historically underrepresented in clinical trials, and female-specific or female-dominant diseases remain substantially underfunded relative to male-dominant diseases.^1^ Similarly, inattention to pelvic symptomatology in women and indiscriminate antibiotic prescribing for nonspecific lower urinary tract symptoms (LUTS) perpetuate gender-based inequities in healthcare and pose significant public health concerns.

## Urinary tract infection as a scapegoat

According to the American Urological Association, the presence of acute-onset urinary symptoms consistent with cystitis (dysuria being the most specific symptom; other symptoms include frequency, urgency, suprapubic pain, and hematuria) is central to the diagnosis of UTI in conjunction with uropathogen detection on urine culture and the absence of vaginal irritation or discharge.^2^ An observational study of 54,000 patients in the ED who were treated with antibiotics for UTI found that only 15% of patients met the diagnostic criteria for UTI.^3^ Similarly, in our hospital system, a review of all outpatient visits coded with UTI as the primary diagnosis revealed that only 17% of patients met these diagnostic criteria. Although more than half of women will be diagnosed with a UTI in their lifetimes,^2^ these data could mean that as many as 80% of these diagnoses are wrong.

## Dismissal of women’s symptoms as “urinary tract infection” results in disproportionate antibiotic burden

Antibiotic overprescription not only is a public health concern but also compromises healthcare equity. As represented in the examples above, female patients, particularly those with pelvic symptoms, frequently experience delays in diagnosis, increased morbidity, and increased healthcare utilization as a result of the indiscriminate attribution of a wide range of symptoms to UTI. The litany of conditions mistaken for UTI, such as undiagnosed neurologic diseases, vulvar lichen sclerosis, labral tears of the hip, irritable bowel syndrome, sacroiliac joint dysfunction, adenomyosis, and myofascial pelvic pain, is endless. In addition to poor recognition of other potentially compromising health conditions, women assume unnecessary risks when antibiotics are inappropriately prescribed. Antibiotic overuse disturbs the commensal microbial flora in the gut and vagina, selecting for antimicrobial-resistant bacteria, increasing susceptibility to *Clostridium difficile* infections, and increasing the risk of vulvovaginal candidiasis, which typically requires additional antifungal treatment. The adverse effects of antibiotics include nausea, diarrhea, rash, vaginal irritation and discharge, and allergic reactions. More and increasingly broad-spectrum antibiotics increase the risk of subsequent sepsis and severe systemic infections, prolong hospital stays, and are associated with increased mortality. These direct effects do not account for the growing body of data suggesting that disturbances in the gut microbiome can have broad consequences on overall health, increasing the risk of obesity, diabetes mellitus, cardiovascular disease, infertility, anxiety and depression, inflammatory diseases, and even cancer.^4^

## Nonmedical logistical and financial pressures may drive poor medical decision-making

Therefore, why must women bear the burden of such healthcare inequities? Undoubtedly, there are various reasons for this: providers’ unfamiliarity with the diagnostic criteria for UTI, lack of awareness of the prevalence of asymptomatic bacteriuria, discomfort with pelvic examinations, fear of accusations of inappropriate behavior, lack of familiarity with confusable conditions, and perpetuated beliefs about the presentations of UTI—all of which we can continue to try to address with education, clinical research, guidelines, best practices, and even artificial intelligence–driven diagnostic decision support tools. However, there is 1 problem that we cannot address with guidelines.

Fundamentally, the profit-driven pressures placed on providers by our current healthcare system may be the most damaging factor. Providers are rewarded for increasing clinical volume, which frequently leads to burnout and reduces the quality of patient-provider interactions. In focus groups exploring women’s experiences with UTI care, patients with recurrent UTI voiced experiences highlighting minimal tangible interactions with a clinician.^5^ Particularly in an emergent care setting, which is often the only setting that can be accessed on short notice, interactions with the clinician were often minimal. Brief histories were taken by a care extender, a few laboratory tests performed, and the patients were sent off after only a short interaction with the treating clinician. The patients are typically not examined and are sent out being told to follow up with a primary care clinician that they likely cannot access for months. Even when seeking treatment from their established physician, the patients usually call an answering service, get told to drop off a urine sample, and end up prescribed an antibiotic, only to have the symptoms continue and the cycle repeat again and again. Worse, if the culture comes back negative, the patients get a voicemail telling them everything is fine. However, the patients still have symptoms, and there is no plan for how to address them.

However, for many physicians, if we do not have time to evaluate a patient and are not comfortable doing so anyway, it may just be survival to slap a diagnosis of UTI on a patient with pelvic symptomatology and send them out to the next provider, hoping the next one will have more time for a more complete evaluation or more expertise. However, the next provider also does not have time, so they look back at other visits, see the previous diagnosis of UTI, and propagate that diagnosis forward. Before long, the patient has had 5 courses of antibiotics, then 10, and then more. Patients become aggravated being caught in a perpetuating cycle of inattention to their pelvic symptoms, so they turn to friends or the Internet for information. On the Internet, the patients find a community of women with “recurrent UTI” who are also angry and frustrated with a medical system that continues to fail them. The patients find burdensome home remedies, restrictive diets, destructive and shameful cleaning rituals, and expensive supplements with no evidence to back them, which rarely improve their condition.

Figuring out the cause of unexplained fluctuating pelvic pain and urinary symptoms in a woman is hard and time-consuming and financially detrimental to physicians who are compensated based on their productivity or monitored for patient turnover. In a recent remodel, one of our primary care offices just removed all the examination tables with stir-ups to facilitate a pelvic examination, as they do not have the time to get patients undressed and positioned for a pelvic examination. However, they still give plenty of antibiotics for UTI. The time has come for us to decide whether we are willing to accept this as a way of providing medical care.

To improve access to care, several automated question-based algorithms for diagnosing UTI are increasingly used to trigger antibiotic prescriptions. These events may occur repeatedly in the community without the necessity of physical examination or culture data. Therefore, women who have taken serial courses of antibiotics for “recurrent UTI” are increasingly reporting to specialists and finding out that they have a UTI-mimicking diagnosis. Although these algorithms may be useful for otherwise healthy women with sporadic acute uncomplicated UTI presentations, they should not be used repeatedly in women with recalcitrant symptoms or for high-risk individuals.^6^, ^7^, ^8^ Guidelines emphasize potential confusable diagnoses, including but not limited to vulvar skin conditions, genitourinary syndrome of menopause, vaginitis, vulvar cysts (eg, Skene gland cysts and Bartholin cysts), pelvic floor myofascial trigger points, pelvic organ prolapse, urethritis, urethral diverticula, urinary retention, and neurologic conditions.^2^ However, these diagnoses only become evident through physical examination findings, culture data, and thoughtful consideration. Furthermore, ongoing symptoms trigger Internet searches, which research has shown to contain significant misinformation and non–evidence-based practices (eg, burdensome cleaning rituals and restrictive diets), further perpetuating this problem.^9^

## Asymptomatic bacteriuria

But the patient has a positive urine culture? Asymptomatic bacteriuria (ASB) is increasingly common as women age, affecting up to 9% of community-dwelling postmenopausal women and up to 50% of female residents of long-term care facilities. The Infectious Diseases Society of America and the American Geriatrics Society recommend against the screening and treatment of ASB in nonpregnant individuals in the absence of any acute localizing UTI symptoms because treatment of ASB does not confer any benefit (does not reduce the risk of death or sepsis) and is associated with harm (adverse effects of antibiotics or antimicrobial resistance). This is true even for functionally impaired adults and older adults living with dementia in the community or long-term care facilities.^10^

## Commitment to evidence-based care

An evidence-based approach to evaluating a patient with LUTS or with any primary complaint would adhere to the definitions and management strategies of UTI and ASB as described in the literature.^2^^,^^10^^,^^11^ In the absence of acute-onset urinary tract symptoms and positive urine culture, antibiotics should not be prescribed for LUTS and certainly not for symptoms unrelated to the urinary tract. Instead, alternative etiologies to explain a patient’s symptoms and presentation should be thoroughly explored through a proper history, physical examination, and ancillary testing as indicated. Familiarity with current guidelines, patient and provider education, use of proper terminology in describing pelvic symptoms and bacteriuria, and a systematic diagnostic approach are the only ways to avoid common misdiagnosis and inappropriate treatment that perpetuate gender-based inequities in women’s health surrounding UTI care.

We advocate a return to the practice of medicine as part of whole-person health, whereby we arrive at an accurate diagnosis only after careful consideration of the full spectrum of information obtained from listening to and examining patients with thoughtful use of urine cultures only for suspected UTI. We must treat the patient, not the test, with a purposeful effort to do better.

## CRediT authorship contribution statement

**Alec Szlachta-McGinn:** Writing – review & editing, Writing – original draft, Conceptualization. **Lynn Stothers:** Writing – review & editing. **A. Lenore Ackerman:** Writing – review & editing, Writing – original draft, Supervision, Resources, Funding acquisition, Conceptualization.
